# Prevalence and risk factors of depression in rural Chinese hemodialysis patients during the COVID-19 pandemic: a multicenter cross-sectional study

**DOI:** 10.3389/fpsyg.2025.1565054

**Published:** 2025-04-03

**Authors:** Zhaoqi Liu, Juhua Lin, Zhiqiang Chen, Rugang Li, Junping Tang, Quan Liu, Lin Ning, Min He

**Affiliations:** Department of Nephrology, Yuebei People's Hospital, Guangdong Medical University, Shaoguan, Guangdong, China

**Keywords:** maintenance hemodialysis, COVID-19 pandemic, depression, rural areas, cross-sectional study

## Abstract

**Purpose:**

This study aimed to assess the prevalence and risk factors of depression among maintenance hemodialysis (MHD) patients in rural China during the COVID-19 pandemic.

**Methods:**

A cross-sectional survey was conducted in 14 hemodialysis centers in northern Guangdong Province from April to October 2021. Depression was evaluated using the Self-Rating Depression Scale. Multivariate logistic regression analysis was employed to identify associated factors.

**Results:**

Of the 450 MHD patients enrolled, 160 (35.6%) met the criteria for depression, with 91.8% cases being of mild severity. After adjusting for demographic, dialysis-related, laboratory, pandemic-associated lifestyle changes, and psychological variables, discomfort during dialysis [Odds ratio (OR) 1.654, 95% Confidence Interval (CI) 1.105–2.474] and infection worry (OR 1.719, 95% CI 1.121–2.636) were significantly associated with an increased risk of depression. In contrast, college education was linked to a lower risk (OR 0.456, 95% CI 0.245–0.846).

**Conclusion:**

During the COVID-19 pandemic in rural China, mild depression were common among MHD patients. Mandatory behavioral interventions did not contribute to depression, while discomfort during dialysis and infection worry emerged as risk factors, and college education was associated with a lower risk.

## 1 Introduction

Maintenance hemodialysis (MHD) is a primary treatment for patients with end-stage renal disease. Due to their reliance on hemodialysis equipment and medical care for survival, these patients often have low immune resistance and frequent complications (Levey et al., 2020; Pecoits-Filho et al., 2020). In addition to suffering numerous physical symptoms, they are prone to emotional disturbances, including a high prevalence of depression. Prior meta-analyses have reported that the prevalence of depressive symptoms in MHD patients is approximately 39.3% when assessed using self-report scales (Palmer et al., 2013). However, recent studies have suggested even higher rates of depression among this population. For instance, a cross-sectional study found that 83.7% of hemodialysis patients exhibited depressive symptoms (Santos et al., 2022). Similarly, another study reported a prevalence of 60.3% for depressive symptoms among MHD patients (Pretto et al., 2020). These findings highlight the significant psychological burden faced by MHD patients and underscore the importance of addressing mental health in this vulnerable population. This vulnerability can be exacerbated during major public health events, potentially leading to an increased incidence of mental health issues.

Current evidence suggests that the COVID-19 pandemic has coincided with a widespread increase in psychiatric disorders (Hossain et al., 2020), warranting the attention of the global health community. To combat the COVID-19 pandemic, various countries and governments have implemented mandatory behavioral non-pharmaceutical interventions, such as restrictions on social interactions and mask-wearing. Although these interventions limit free communication, potentially exacerbating depression, compliance with them can make individuals feel safe and help alleviate depressive symptoms. Thus, these measures are a “double-edged sword”: while they help control the spread of the virus and provide a sense of safety, they can also exacerbate mental health issues like depression and anxiety by limiting social interactions and increasing feelings of isolation (Perlis et al., 2023).

Considering the health vulnerability of the maintenance hemodialysis (MHD) population, it is generally believed that pandemic-associated psychological stress will have a depressive impact on MHD patients (Lee et al., 2020; Hao et al., 2021; Mueller et al., 2021; Askaryzadeh Mahani et al., 2023; Ibrahim et al., 2023; Koşunalp and Kavurmaci, 2023; Oviedo Flores et al., 2023; Shahrbabaki et al., 2023). However, studies on dialysis populations have shown inconsistent results. Some research, primarily from countries with advanced healthcare systems, suggests that the pandemic has not significantly affected the mental health of dialysis patients (Bonenkamp et al., 2021; Nadort et al., 2022). Other findings focus on mandatory behavioral interventions and indicate that these measures do not impact mental health (Jones et al., 2024). A large-scale epidemiological survey from South Korea found that not wearing masks indoors was most strongly associated with depression; those who did not adhere to public health measures were more likely to experience depression (Byun et al., 2022).

Given their existing health vulnerabilities, the pandemic has introduced additional layers of complexity for these patients. In addition to their weakened immune systems and susceptibility to complications (Levey et al., 2020; Pecoits-Filho et al., 2020), their frequent hospital visits and prolonged exposure to healthcare settings place them at a higher risk of infection (Government Accountability Office, 2010). This vulnerability is particularly pronounced during public health emergencies like the COVID-19 pandemic. Moreover, rural MHD patients often face unique challenges during the pandemic. Limited medical resources and access to healthcare in rural areas can further complicate their treatment and management (Ngo, 2022). Additionally, the psychological burden of living with a chronic disease, combined with the stress of the pandemic, can have a significant impact on their mental health. Studies have shown that the COVID-19 pandemic has led to increased anxiety and depression among patients with chronic illnesses, particularly those undergoing regular medical treatments like hemodialysis (Chen et al., 2022). Despite these risks, there is currently a lack of research examining the causal relationship between the pandemic and mental health outcomes in rural MHD patients. While some studies have suggested that the pandemic may have exacerbated mental health issues in this population, the evidence remains limited and inconsistent (Bonenkamp et al., 2021; Nadort et al., 2022). This gap in knowledge highlights the need for further investigation into the specific factors contributing to depression and other mental health problems among rural MHD patients during the COVID-19 pandemic.

This study aims to investigate the depression prevalence and analyze the association between demographic factors, medical parameters, pandemic-associated lifestyle changes, and depression, thereby exploring the risk factors for depression among the rural MHD population. It addresses the gap in understanding the impact of the pandemic on the mental health of rural MHD patients and provides insights to improve their clinical care.

## 2 Methods

Our study was designed as a multicenter, cross-sectional survey incorporating a prospective questionnaire approach to assess the prevalence and risk factors of depression among MHD patients in rural China during the COVID-19 pandemic.

### 2.1 Patient inclusion

Patients from 14 hemodialysis centers in the northern rural areas of Guangdong Province were selected for this study, conducted from April 2021 to October 2021. The inclusion criteria were as follows: (1) undergoing hemodialysis for 6 months or more; (2) aged 18 years or older; (3) fully conscious and able to complete the questionnaire independently or with assistance; (4) no serious complications in the past 1 month. The serious complications refer to any of the following conditions: complications or comorbidities that require emergency department visits or hospitalization; existing heart failure, as determined by a physician. Exclusion criteria involved patients who were unwilling to participate.

### 2.2 Ethical issues

The study protocol was reviewed and approved by the hospital’s Ethics Committee. All patients and their family members were informed about the study’s details and voluntarily signed the informed consent.

### 2.3 Data collection

Objective clinical data were extracted from the hemodialysis management software system and verified by the attending nephrologists. The following clinical parameters were collected: (1) Demographic information (age, sex, education level, marital status, and monthly family income); (2) Nutritional status (serum albumin levels) and anemia indicators; (3) Dialysis details (years on dialysis, frequency of sessions, single-session fluid removal volume and duration). Subjective data, including symptoms experienced during dialysis and pandemic-related information (daily masking duration, discussions with other patients about the pandemic, understanding of pandemic updates), were collected using a locally-designed recording forms administered by trained hemodialysis nurses.

The Self-Rating Depression Scale (SDS), a widely used 20-item self-reported questionnaire, was utilized to assess depression. Upon obtaining informed consent, SDS questionnaires were distributed. Nurses, who were uniformly trained, explained the survey content and the instructions for completing the questionnaire, ensuring patients could complete it independently or with assistance. The SDS scores range from 25 to 100, with a score of 50 or above indicating clinical depression: 50–59 signifies mild depression, 60–69 moderate, and 70 or above severe (Zung, 1965; Dunstan and Scott, 2019).

To ensure the consistency and accuracy of data collection, nurses at each center were uniformly trained on how to administer the SDS questionnaire and complete the locally-designed recording forms. This training included instructions on how to explain the survey content to participants and how to record their responses accurately.

### 2.4 Statistical methods

Because the rapidly evolving nature of the COVID-19 pandemic and the logistical constraints in accessing patients across multiple rural centers made it impractical to strictly follow a pre-calculated sample size, we adopted a pragmatic approach by including all eligible MHD patients from 14 hemodialysis centers in rural northern Guangdong Province over the study period (April to October 2021). This approach aimed to capture a comprehensive snapshot of the patient population during the pandemic and ensure that our sample was as representative as possible of the rural MHD population in this region.

Statistical analyses were performed using SPSS software version 26.0. Initial comparisons between depressed and non-depressed groups were conducted using independent sample *t*-tests for continuous variables and chi-square tests for categorical data. For the t-tests, we verified the assumptions of normality and homogeneity of variances using Shapiro–Wilk tests and Levene’s tests, respectively. Univariate analyses were also conducted to screen the data and evaluate the individual associations of each variable with depression. Significant variables from these comparisons were then included in a Wald stepwise multivariate logistic regression analysis to identify independent risk factors for depression, adjusting for demographic, medical, and pandemic-related variables. The logistic regression model was assessed for fit using the Hosmer-Lemeshow goodness-of-fit test, which evaluates how well the model fits the observed data. Odds ratios (OR) and their 95% confidence intervals (CI) were calculated to quantify the strength and significance of the associations between the predictors and depression. The confidence intervals were constructed using the Wald statistic, which provides an estimate of the precision of the odds ratios. All statistical tests were two-tailed, with a significance level set at *p* < 0.05.

## 3 Results

### 3.1 Clinical characteristics and prevalence of depression

A total of 450 hemodialysis patients completed the survey, of which 238 (52.9%) were male, with an average age of 54.9 ± 13.9 years. The overall SDS score was 45.1 ± 8.8, with emotional and psychological symptoms scoring 7.3 ± 2.3, physical and behavioral symptoms 17.7 ± 3.6, and social and cognitive symptoms 20.1 ± 4.9.

Among the 450 patients, 160 were identified as having depression, including 147 (91.8%) with mild, 11 (6.9%) with moderate, and 2 (1.3%) with severe depression. The clinical characteristics of patients with or without depression are summarized in Table 1. Compared to non-depressed individuals, those with depression were less educated and had lower incomes, and more frequently self-reported worry about COVID-19 infection. No significant differences were found in laboratory indicators or in lifestyle during the pandemic (Table 1).

**Table 1:** Clinical characteristics of patients without and with depression.

	Non-depressed *N* = 290	Depressed *N* = 160	*t*/*χ*^2^	*p* value
Demographic indicators				
Age	54.5 ± 14.2	55.6 ± 13.4	−0.849	0.396
Male [*n* (%)]	160 (55.2%)	78 (48.8%)	1.707	0.191
College education	60 (21.3%)	15 (9.6%)	9.624	0.002
Marriage			1.772	0.621
Unmarried	37 (12.8%)	14 (8.8%)		
Married	225 (77.6%)	130 (81.3%)		
Divorced	12 (4.1%)	6 (3.8%)		
Widowed	16 (5.5%)	10 (6.3%)		
Monthly income ≥5, 000 yuan	127 (43.8%)	44 (27.5%)	11.618	<0.001
Dialysis-related parameters and symptoms				
HD vintage [*n* (%)]			0.556	0.757
<1 year	67 (23.1%)	42 (26.3%)		
1–<3 year	55 (19.0%)	29 (18.1%)		
≥3 year	168 (57.9%)	89 (55.6%)		
HD frequency [*n* (%)]			1.654	0.437
≤Twice a week	56 (19.3%)	32 (20.0%)		
Five times every 2 weeks	219 (75.5%)	115 (71.9%)		
Three times a week	15 (5.2%)	13 (8.1%)		
Discomfort during dialysis [*n* (%)]	121 (41.7%)	89 (55.6%)	8.005	0.005
Laboratory parameters				
Hemoglobin			3.679	0.159
<90 g/L	66 (22.8%)	45 (28.1%)		
90–129 g/L	212 (73.1%)	104 (65.0%)		
≥130 g/L	12 (4.1%)	11 (6.9%)		
Serum albumin			4.744	0.192
<30 g/L	15 (5.2%)	14 (8.8%)		
30–34 g/L	68 (23.4%)	43 (26.9%)		
35–39 g/L	160 (55.2%)	73 (45.6%)		
≥40 g/L	47 (16.2%)	30 (18.8%)		
Lifestyle change				
Daily masking time [*n* (%)]			6.004	0.050
<2 h	100 (34.5%)	41 (25.6%)		
2–<4 h	61 (21.0%)	48 (30.0%)		
≥4 h	129 (44.5%)	71 (44.4%)		
Mask discomfort [*n* (%)]	96 (33.1%)	67 (41.9%)	3.434	0.064
Psycho well-being				
Daily concern for pandemic [*n* (%)]	212 (73.1%)	126 (78.8%)	1.759	0.185
Reduce outdoor activities [*n* (%)]	183 (63.1%)	106 (66.3%)	0.444	0.505
Infection worry [*n* (%)]	166 (57.2%)	113 (70.6%)	7.839	0.005

### 3.2 Factors influencing depressive severity

**Figure 1:**
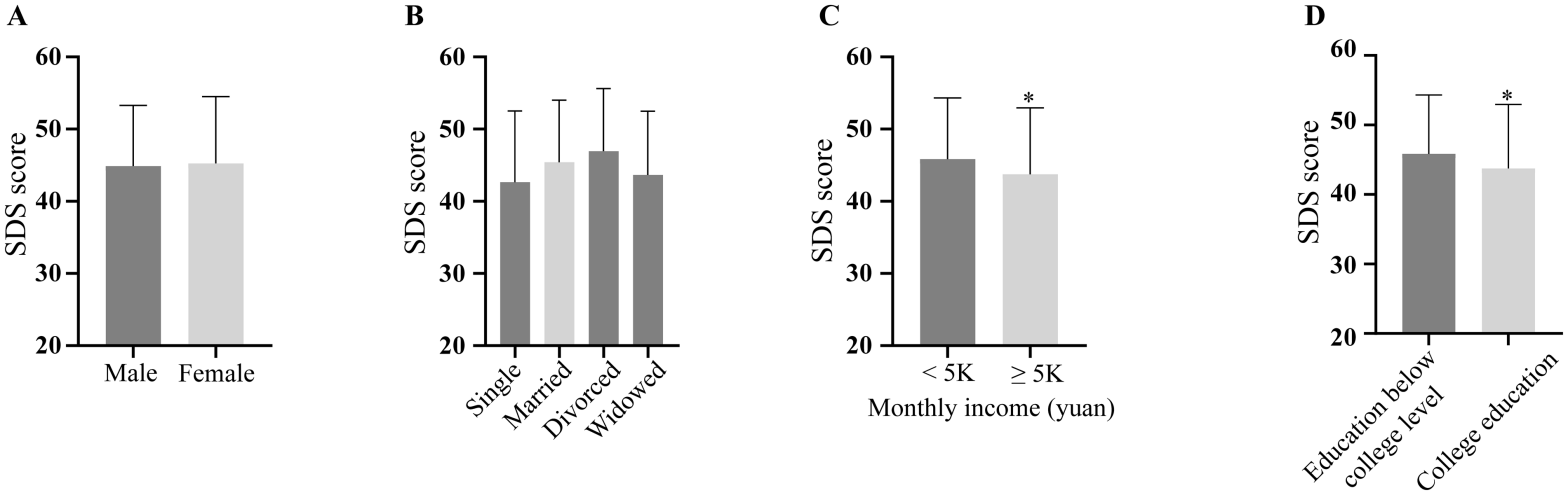
Depression severity scores across clinical subgroups. SDS: self-rating depression scale.

Given the predominance of mild depression among patients, we utilized the SDS scores to assess its severity across clinically distinct subgroups. Demographic analyses revealed no significant differences in SDS scores based on sex or marital status (Figures 1A,B), but higher educational levels and monthly incomes correlated with lower SDS scores (Figures 1C,D), indicating an education-income link with depression.

**Figure 2:**
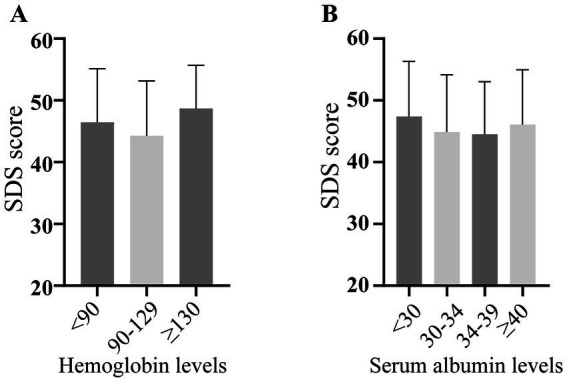
Depression severity scores across different laboratory status. SDS: self-rating depression scale.

**Figure 3:**
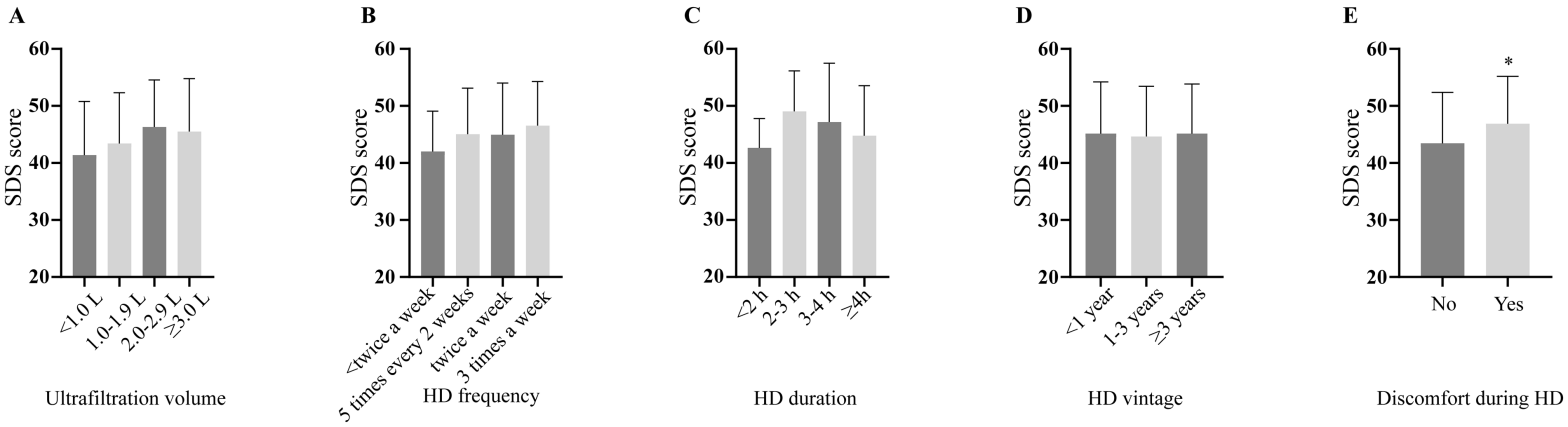
Depression severity scores according to different dialysis parameters. SDS: self-rating depression scale.

SDS scores were unaffected by laboratory results and dialysis parameters, with no variations observed for serum albumin, hemoglobin levels (Figures 2A,B), or dialysis specifics (volume, frequency, session duration, years on dialysis or discomfort during dialysis; Figures 3A–E).

**Figure 4:**
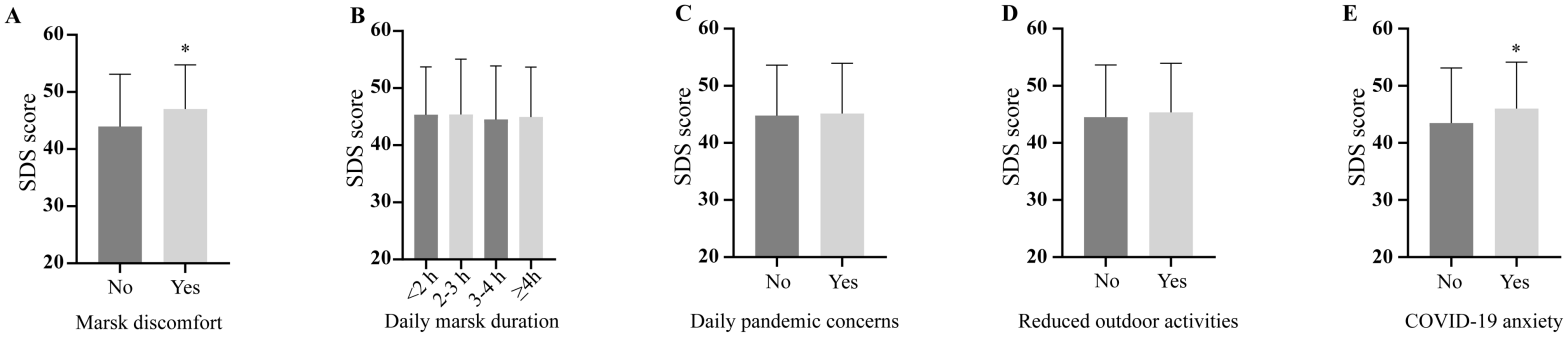
Depression severity in different lifestyle groups based on the COVID-19 Pandemic. SDS: self-rating depression scale.

Patients experiencing mask discomfort had higher SDS scores than those without (Figure 4A), while mask duration did not impact scores significantly (Figure 4B), indicating subjective emotional responses play a role in mask-related distress. Other lifestyle changes due to the pandemic, such as daily pandemic concerns and reduced outdoor activities, did not influence SDS scores (Figures 4C,D). However, anxiety about COVID-19 infection was associated with higher SDS scores (Figure 4E), emphasizing personal psychological factors’ impact on depression over routine activity modifications.

Taken above at all, individuals with low income, limited education, mask discomfort, and heightened COVID-19 anxiety are at increased risk for depression, highlighting the need for integrated psychological and physical health support, especially during crises.

### 3.3 Depression-associated risk factors

We then further analyzed the variables listed in Table 1 that showed statistical significance to investigate their independent associations with depression. The analysis included demographic variables (age, sex, education level, marital status, monthly family income), dialysis-related parameters (dialysis vintage, frequency of sessions, discomfort during dialysis), and pandemic-related factors (infection worry, mask discomfort, daily concern for the pandemic, and reduced outdoor activities). Given a strong association observed between education and income in preliminary analysis (data not shown), only education was included in the modeling as a potential variable. After adjusting for multiple factors, three variables remained significant in the model: college education was associated with a lower risk of depression [OR 0.456 (95% CI: 0.245–0.846)], while discomfort during dialysis [OR 1.654 (95% CI: 1.105–2.474)] and infection worry [OR 1.719 (95% CI: 1.121–2.636)] were linked to an increased risk of depression (Table 2).

**Table 2:** Independent risk factors for depression.

Variables	Odds ratio	95% confidential interval	*p* value
College education	0.456	0.245–0.846	0.005
Discomfort during dialysis	1.654	1.105–2.474	0.014
Infection concern	1.719	1.121–2.636	0.013

## 4 Discussion

This study provided the epidemiological characteristics of depression in 450 MHD patients during the COVID-19 pandemic in rural areas of northern Guangdong Province, China, and found that about 1/3 of the patients met the criteria for depression with SDS scores of more than 50. After univariate and multivariate analysis, we found that discomfort during dialysis, and infection worry were risk factors associated with depression. Higher education was associated with a lower risk of depression.

This study adopts an SDS score > 50 as the criterion for depression and reveals that the incidence of depression among MHD patients in rural China during the pandemic era is 35.6%. This study adopts an SDS score > 50 as the criterion for depression and reveals that the incidence of depression among MHD patients in rural China during the pandemic era is 35.6%. This rate is similar to the pre-pandemic rates reported in previous studies using the Patient Health Questionnaire-9 (PHQ-9) for depression assessment among hemodialysis patients (Zhang et al., 2024; Feng et al., 2022). Given the weak correlation between PHQ-9 and SDS scores in the general population, with a mere 0.29 correlation coefficient (Wang et al., 2014), no direct conversion between these measures is feasible. Conversely, the depression detection rate of 35.6% during the pandemic in our study is lower than the pre-pandemic rates reported in rural MHD populations using other assessment tools. For example, Teles et al. and Norozi et al. both used the Beck Depression Inventory (BDI) and reported detection rates of 42.7 and 44.8%, respectively (Teles et al., 2014; Norozi Firoz et al., 2019). Additionally, Al-Jabi et al. used the Beck Depression Inventory-II (BDI-II) and found a higher detection rate of 73% (Al-Jabi et al., 2018). These findings highlight that studies employing distinct criteria for depression assessment are not directly comparable, and it is challenging to draw definitive conclusions about whether the pandemic has increased depression rates in the dialysis population. However, Ibrahim et al. used the BDI during the pandemic and found that 66.2% of their MHD sample exhibited depressive symptoms, with 61.4% meeting diagnostic criteria for depression (Ibrahim et al., 2023). Comparing these results with pre-pandemic studies using the BDI (Teles et al., 2014; Norozi Firoz et al., 2019) suggests that the pandemic may have significantly increased depression rates in this population. Another key factor contributing to the discrepancy in reported depression rates is the rural setting of our investigation, which contrasts with the non-rural areas studied by Hao W et al. Using identical depression assessment criteria, they reported a depression detection rate of 32.1% among 321 hemodialysis patients, slightly lower than our findings (Hao et al., 2021). This difference may be attributed to demographic variations between the two studies, such as a higher proportion of our subjects with monthly income lower than 5,000 yuan (62% compared to 53%) and dialysis vintage more than a year (75.8% versus 67.0%).

Our study focused on the association between pandemic-associated lifestyle changes and depression. We observed a trend toward an increased depression rate among patients who wore masks for longer periods; however, this increase was not statistically significant when compared to those without depression. Additionally, mask discomfort was not associated with depression. These results suggest that mask-wearing itself does not elevate the risk of depression. After adjusting for multiple variables, the mask-related index remained unassociated with depression. Similarly, other lifestyle changes, such as daily concerns about the pandemic and reduced outdoor activities, were also not linked to depression. These findings support the notion that mandatory behavioral interventions do not contribute to depression in the MHD population in rural areas of China. In addition to the key factors associated with depression identified in our study—such as discomfort during dialysis, infection-related worries, and lower levels of education—other risk factors have been documented in the literature, including physical health status, psychological stress, social support, and the impact of the COVID-19 pandemic. Previous research has shown that physical discomfort and psychological stress are significant contributors to depression in this population (Zhang et al., 2024; Feng et al., 2022). Additionally, the COVID-19 pandemic has exacerbated existing mental health challenges by introducing additional stressors, such as infection fears and lifestyle disruptions, which are particularly impactful for vulnerable populations like MHD patients (Perlis et al., 2023). Considering that these factors have been extensively studied in prior research and adhering to the principle of simplicity in questionnaire design, our study did not include these additional factors in the analysis. However, their importance should not be overlooked in future research and clinical practice.

Our study has several limitations that should be acknowledged. First, the binary measure of reduced outdoor activities may not fully capture the complexity of lifestyle changes during the pandemic, partly due to lower education levels in rural areas that hinder detailed data collection. Second, incomplete clinical databases in some centers limited our ability to include all relevant laboratory data and comorbidities in the analysis. This may have affected the comprehensiveness of our results. Third, our study relied on self-reported data for depression assessment using the SDS, which may be subject to reporting bias. Future studies could consider using additional diagnostic tools or interviews to validate depression diagnoses. Fourth, the cross-sectional design of our study limits our ability to establish causality between identified risk factors and depression. Longitudinal studies would be beneficial to better understand the temporal relationships between these variables. Finally, this cross-sectional survey was conducted from March to October 2021, a period that corresponds to the national vaccination campaign stage following the shift from the first large-scale outbreak to sporadic outbreaks in mainland China. Therefore, the interpretation of the study’s findings should take into account the sociological context of this specific timeframe. Our study was conducted in a specific rural region of China, which may limit the generalizability of our findings to other regions or populations with different healthcare systems and cultural contexts. Despite these limitations, our multicenter, prospective design is a notable strength, particularly given the challenges of data collection in rural areas during the pandemic.

The multicenter and prospective design is a notable strength of this study, particularly given the difficulties in acquiring data from rural areas during the pandemic. Importantly, the three risk factors for depression that were identified in our research are easily recognizable, thereby highlighting the feasibility of reproducing these results within clinical contexts. Our findings offer persuasive evidence to guide intervention strategies for this demographic in future public health crises.

Our study emphasizes the importance of integrating mental health support into routine care for MHD patients, especially during public health emergencies. Healthcare systems should consider regular mental health screenings and targeted interventions to address identified risk factors. Public health strategies should prioritize education and communication to reduce infection-related anxiety and promote resilience (Perlis et al., 2023). This is particularly important in rural areas where access to mental health resources is often limited.

In conclusion, during the COVID-19 pandemic in rural China, mild depression were common among MHD patients. Mandatory behavioral interventions did not contribute to depression, while discomfort during dialysis and infection worry emerged as risk factors, and college education was associated with a lower risk.

## Data Availability

The raw data supporting the conclusions of this article will be made available by the authors, without undue reservation.
